# Soluble Tau has devastating effects on the structural plasticity of hippocampal granule neurons

**DOI:** 10.1038/s41398-017-0013-6

**Published:** 2017-12-08

**Authors:** M Bolós, N Pallas-Bazarra, J Terreros-Roncal, JR Perea, J Jurado-Arjona, J Ávila, M Llorens-Martín

**Affiliations:** 1 Department of Molecular Neuropathology, Centro de Biología Molecular “Severo Ochoa”, CBMSO, CSICUAM, Madrid, Spain; 2 Network Center for Biomedical Research on Neurodegenerative Diseases (CIBERNED), Madrid, Spain; 3 Department of Molecular Biology, Faculty of Sciences, Universidad Autónoma de Madrid, Madrid, Spain

## Abstract

Tau is a neuronal microtubule-associated protein with countless physiological functions. Although the detrimental effects of insoluble aggregated Tau have been widely studied, recent evidence supports the notion that soluble Tau (composed mostly of monomers and dimers) is also toxic for neurons. Here we evaluated the long-term impact of a single stereotaxic injection of human soluble Tau on hippocampal granule neurons in mice. At the ultrastructural level, soluble Tau reduced the number of afferent synapses and caused a dramatic depletion of synaptic vesicles both in afferent and efferent synapses. Furthermore, the use of an RFP-expressing retrovirus revealed that soluble Tau altered the morphology of newborn granule neurons and reduced their afferent (dendritic spines) and efferent (mossy fiber terminals) connectivity. Finally, soluble Tau caused specific impairment of behavioral pattern separation capacity. Our results thus demonstrate for the first time that soluble Tau causes long-term detrimental effects on the morphology and connectivity of newborn granule neurons and that these effects correlate with impaired behavioral pattern separation skills. These data might be relevant for the field of neurodegenerative disorders, since they contribute to reinforcing the pathological roles played by distinct Tau species *in vivo*.

## Introduction

Tau is a neuronal microtubule-associated protein (MAP) that plays a central role in microtubule stabilization^1^. The relevance of Tau during brain development has been addressed extensively^2^. Moreover, recent evidence points to novel roles played by Tau during adult neurogenesis^3^. Tau regulates not only microtubule stability but also axonal transport^4^ and it plays key roles at synapses^5,6^. During the human lifespan, Tau protein expression and function are intricately regulated by alternative splicing and post-transcriptional modifications^7–9^.

A group of neurodegenerative diseases known as Tauopathies is characterized by alterations in Tau metabolism. In these disorders, Tau usually losses solubility and tends to form aggregate structures that impair cell function and trigger neuronal cell death and neurodegeneration^9,10^. Current hypotheses postulate that Tau is released to the extracellular space and propagates from one cell to another following a stereotypic pattern of spread among brain regions^11–15^ and that the presence of this extracellular Tau damages neighboring neuronal and glia cells. It is also believed that the complete aggregation and insolubilization of Tau is not required for this protein to trigger neurodegeneration^16^ and that small oligomers of Tau are highly toxic for neurons^13^. However, the *in vivo* neurotoxic potential of soluble Tau has not been fully elucidated to date.

The hippocampus is one of the brain regions most affected in Tauopathies^17^. Moreover, synaptic transmission to and from the dentate gyrus (DG) is a crucial stage for information processing within the hippocampal trisynaptic circuit^18,19^. Given these considerations, we aimed to explore whether the presence of soluble Tau (composed mostly of monomers and dimers) has long-term effects on the structural plasticity of the predominant neuronal population present in the DG, namely hippocampal granule neurons. Of note, the adult DG has several unique anatomical and functional features. One of the most relevant is adult hippocampal neurogenesis (AHN)^20^, a process that encompasses the generation, maturation, and synaptic integration of newborn granule neurons into the hippocampal trisynaptic circuit. This process is impaired in animal models of Tauopathies and patients with the same conditions, Alzheimer disease (AD) being one^21–23^. These data are in agreement with the relevant role played by these newly generated granule neurons in certain aspects of hippocampal-dependent learning, such as behavioral pattern separation^24–26^. Noteworthy, these cognitive skills are profoundly impaired in patients with Tauopathies^27,28^. In the present study, we show that the stereotaxic injection of soluble Tau into mice causes long-term alterations in the structural plasticity of granule neurons and that these alterations correlate with selective impairment of behavioral pattern separation ability.

## Materials and methods Section heading Material and methods changed to Materials and methods, please confirm. OK

### Animals

Six-week-old female C57Bl/6J Ola Hsd mice were purchased from EnVigo Laboratories, Spain. Mice were housed in a specific pathogen-free colony facility, in accordance with European Community Guidelines (directive 86/609/EEC), and handled following European and local animal care protocols. Animal experiments received the approval of the CBMSO’s (AEEC-CBMSO-23/172) and national (PROEX 205/15) Ethics Committee. Animals were left undisturbed for 2 weeks before being subjected to experimental manipulation.

### Experimental design

**Fig. 1:**
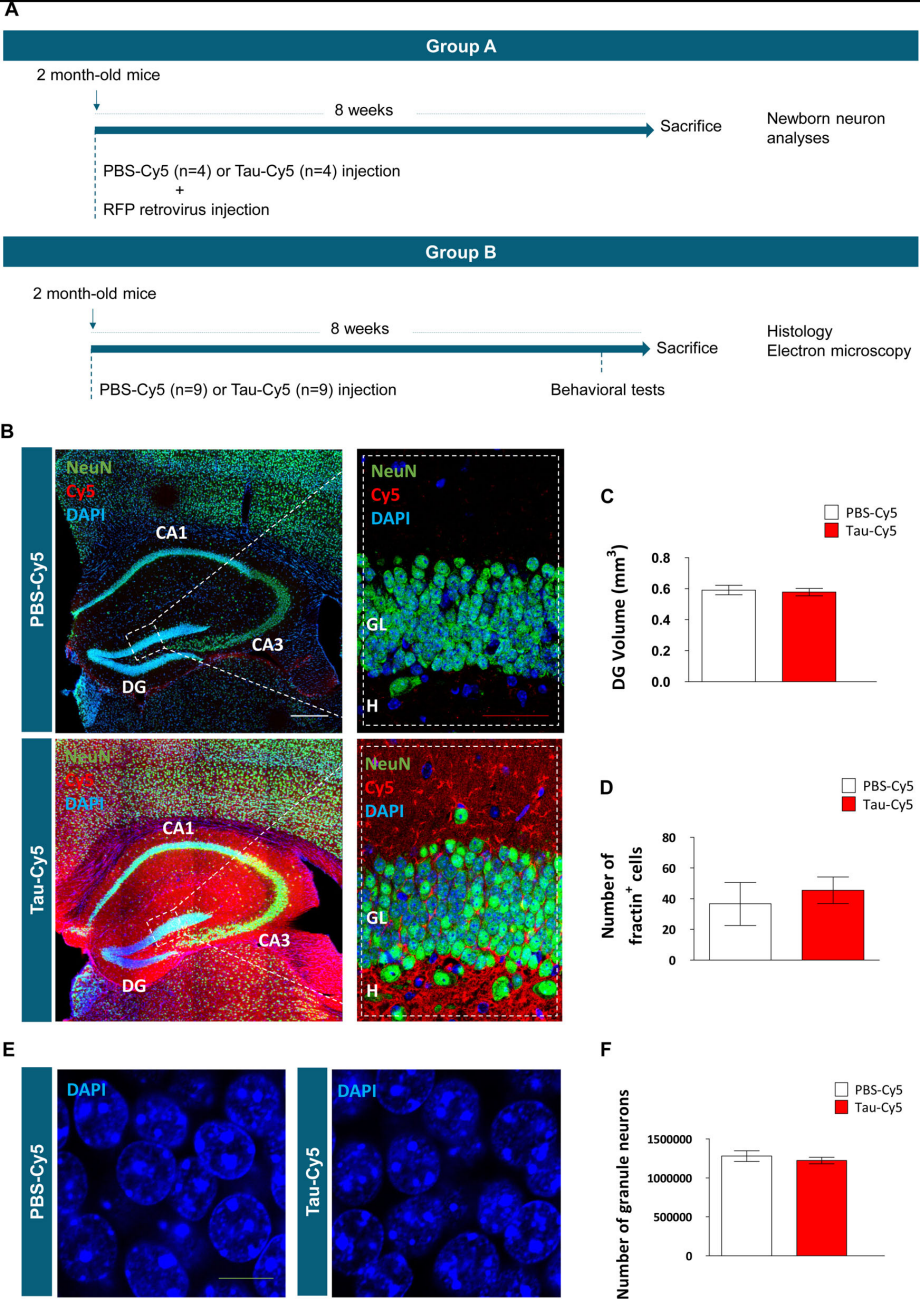
The presence of soluble Tau-Cy5 does not alter the gross morphology of the DG (**a**) Schematic diagram of the experimental design. Animals received a stereotaxic injection of either Tau-Cy5 (13) or PBS-Cy5 (13) in the hippocampal dentate gyrus (DG). Of these animals, eight (four Tau-Cy5 and four PBS-Cy5) also received an injection of an RFP-expressing retrovirus in order to label newborn granule neurons (group A). The remaining 18 animals (9 Tau-Cy5 and 9 PBS-Cy5) formed the group B. Of these animals, six (three Tau-Cy5 and three PBS-Cy5) were used for electron microscopy analyses. Animals were sacrificed 8 weeks after stereotaxic injections. (**b**) Representative tile-scan images of the whole hippocampus of PBS-Cy5- and Tau-Cy5-injected mice. An intense Cy5 signal can be observed 8 weeks after Tau-Cy5 injection, but not after PBS-Cy5 injection. (**c**) Quantification of DG volume in PBS-Cy5- and Tau-Cy5-injected mice. (**d**) Number of apoptotic fractin^+^ cells in the DG. (**e, f**) Representative images (**e**) and quantification (**f**) of the number of mature granule neurons in PBS-Cy5- and Tau-Cy5-injected mice. Tau-Cy5 injection did not lead to differences in any of these general morphometric parameters. In (**d**, **f**) graphs represent mean ± SEM; *n* = 6 mice per experimental condition. DG, dentate gyrus; GL, granular layer; H, hilus. White scale bar: 300 µm. Red scale bar: 50 µm. Green scale bar: 10 µm

Animals were randomly assigned to one of two groups, namely PBS-Cy5- or Tau-Cy5-injected. A group of eight mice (4 PBS-Cy5 and 4 Tau-Cy5) was used for retroviral injections (group A). A group of 18 mice (9 PBS-Cy5 and 9 Tau-Cy5) was used for behavioral, histological, and electron microscopy determinations (group B). Of the latter group, three PBS-Cy5- and three Tau-Cy5-injected animals were used for electron microscopy determinations. A schematic experimental design is shown in Fig. 1. Animals were subjected to stereotaxic injections at 2 months of age (red fluorescent protein (RFP)-expressing retrovirus plus either PBS-Cy5 (4) or Tau-Cy5 (4) in the case of group A, and only PBS-Cy5 (9) or Tau-Cy5 (9) in the case of group B). Animals were sacrificed 2 months after stereotaxic injections. Animals belonging to group B received 5-Iodo-2′-deoxyuridine (IdU) at 1 month of age. Behavioral tests were performed on animals belonging to group B immediately before sacrifice.

### Tau-Cy5

The recombinant human Tau isoform containing two N-terminal inserts and four microtubule binding repeats (Tau42^7^) was isolated as described previously^29^. Briefly, the proteins isolated were characterized by gel electrophoresis, followed by staining with Coomassie blue. Purified soluble Tau, which is mainly composed of monomers and dimers (Supplementary Figure S1), was labeled with sulfoindocyanine Cy5 dye (GE Healthcare, UK), as described previously^30^ and following the manufacturer’s recommendations. Briefly, 1 ml of recombinant Tau protein (2 mg/ml) was mixed with a sample of Cy5 dye at room temperature for 1 h to ensure binding to the fluorophore. To remove excess free dye, the mixture was dialyzed overnight at 4°C against phosphate-buffered saline (PBS) and filtrated through a Sephadex G-50 column. Cy5-coupled Tau protein was characterized by western blotting with an anti-Cy5 antibody (Abcam; 1:1000).

### Ultracentrifugation

#### Sedimentation velocity (SV) assay

About 0.5 mg/ml of Tau42 in PBS (pH 7.4) were loaded (320 ml) into analytical ultracentrifugation cells. The experiments were carried out at 48 krpm in an XLI analytical ultracentrifuge (Beckman-Coulter Inc.). Sedimentation coefficient distributions were calculated by least-squares boundary modeling of SV data using the continuous distribution *c* (*s*) Lamm equation model as implemented by SEDFIT 15.01b^31^. Experimental *s* values were corrected to standard conditions (water, 20°C, and infinite dilution) using the program SEDNTERP^32^ to get the corresponding standard sw values (s20,w).

#### Sedimentation equilibrium assay

Using the same experimental conditions as in the SV experiments, short columns (90 ml) Sedimentation equilibrium experiments were carried out at speeds ranging from 7000 to 10,000 rpm. After the last equilibrium scan, a high-speed centrifugation run (48,000 rpm) was done to estimate the corresponding baseline offsets. Weight-average buoyant molecular weights of protein were determined by fitting a single species model to the experimental data using the HeteroAnalysis program^33^, and corrected for solvent composition and temperature with the program SEDNTERP^32^.

### Retroviral stock preparation

We used a retroviral stock encoding for RFP^34^. The plasmids used to produce the virus were kindly provided by Prof. FH. Gage and Dr. F. Calegari. Retroviral stocks were concentrated to working titers of 1 × 10^7^–2 × 10^8^ pfu/ml by ultracentrifugation^35^. Since the retroviruses used are engineered to be replication-incompetent, only cells dividing at the time of surgery are infected^35^. In the DG, these proliferative cells are almost exclusively restricted to newborn granule neurons^35^.

### Stereotaxic surgery

Mice were anesthetized with isoflourane and placed in a stereotaxic frame. Retroviruses were injected into the DG at the following coordinates (mm) relative to bregma in the anteroposterior, mediolateral, and dorsoventral axes: [−2.1, ±1.4, 2.2]. Either PBS-Cy5 or Tau-Cy5 was injected into the DG at the following coordinates (mm) relative to bregma in the anteroposterior, mediolateral, and dorsoventral axes: [−1.6, ±1.4, 2.2]. Two microliters of retrovirus, PBS-Cy5 or Tau-Cy5 solution was infused at a rate of 0.2 µl/min via a glass micropipette. To avoid any suction effect of the solution injected, micropipettes were kept in place at the site of injection for an additional 5 min before being slowly removed. All animals were 8 weeks old at the time of injection.

### Administration of the thymidine analog IdU

The thymidine analog IdU (Sigma‐Aldrich) was used to analyze the survival of 12‐week‐old newborn granule neurons. IdU was administered over 24 h diluted in drinking water at 0.92 mg/ml. These doses were based on equimolar doses of 0.8 mg/ml BrdU^36^.

### Behavioral tests

- *Elevated Plus Maze (EPM) test:* animals were tested in a 5-min single trial of the EPM (Cibertec, Madrid, Spain) paradigm, as a measurement of anxiety-like behavior, as previously described^37^. Briefly, mice were allowed to move freely along the apparatus under constant intense white light. Animal movement was video-recorded using Anymaze (Stoelting, USA) software and analyzed automatically by the software. The total time spent in the open arms and the total distance covered are shown in the graphs.
- *Open Field (OF) and Novel Location Preference (NLP) tests*: animals were placed in a square (45 × 45 cm), constantly illuminated, open-field methacrylate arena. The test was performed on three consecutive days, on which animals were subjected to a single 10-min trial. On the first day, they were placed inside the arena and allowed to explore it (habituation) (OF phase). During the second day (sample phase), two identical objects were placed symmetrically in the central part of the arena. On the third day (test phase), one of the objects (newly located object) was moved to a peripheral position, while the other one remained in its original position. Animal performance was video-recorded using Anymaze (Stoelting, USA) software and analyzed automatically by the software. Total distance moved, speed, time immobile, number of immobile episodes, entries to the center and time in the center on day 1 are shown in the graphs. Memory index (percentage of time exploring the novel-located object/ total time exploring novel + unaltered object) on day 3 is shown in the graphs.

### Sacrifice

Mice were fully anesthetized with an intraperitoneal pentobarbital injection (EutaLender, 60 mg/kg). Animals belonging to group A were transcardially perfused with 0.9% saline followed by 4% paraformaldehyde in phosphate buffer. Brains were removed and post-fixed overnight in the same fixative. Animals belonging to the group B were perfused only with 0.9% saline. Brains were removed, and left and right hemispheres were separated. Left hemispheres were used for biochemical analyses. Hippocampi were quickly dissected on ice and frozen in dry ice (*n* = 9 animals per experimental group). Right hemispheres were used for histological or electron microscopy analyses. For histological determinations, right hemispheres of six animals belonging to each experimental group were immersed in 4% paraformaldehyde overnight. For electron microscopy determinations, the right hemisphere of the three remaining animals belonging to each experimental group was immersed in 4% paraformaldehyde + 2% glutaraldehyde in phosphate buffer overnight.

### Immunohistochemistry

Sagittal brain sections were obtained on a Leica VT1200S vibratome (50-µm-thick sections). For immunohistochemical analysis, series of brain slices were randomly made up of one section from every ninth. Slices were initially pre-incubated in phosphate buffer with 1% Triton X-100 and 1% bovine serum albumin, and dual immunohistochemistry was then performed as described previously^23^, using the following primary antibodies: rabbit anti-RFP (Millipore, 1:2000); mouse anti-Cy5 (Abcam, 1:500); goat anti-Doublecortin (DCX) (Santa Cruz, 1:500); rabbit anti-Fractin (a fragment of actin cleaved by caspase 3) (BD Biosciences, 1:500); mouse anti-BrdU/IdU (BD Biosciences, 1:500); rabbit anti-MAP-2 (Synaptic Systems, 1:500); and rabbit anti-NeuN (Millipore, 1:1000). To detect the binding of primary antibodies, Alexa-488 donkey anti-mouse and Alexa-555 donkey anti-rabbit (Invitrogen, 1:1000) secondary antibodies were used. All the sections were counterstained for 10 min with DAPI (Merck, 1:5000) in order to label nuclei.

### Electron microscopy

After a fixation step, 200-µm sagittal sections were obtained on a Leica VT1200S vibratome. Four sections per mouse containing the whole hippocampus were post-fixed in 2% osmium tetroxide (OsO_4_) for 2 h. They were then rinsed, dehydrated, and embedded in Durcupan (Durcupan; Fluka). Serial semi-thin sections (1 µm) were cut with a diamond knife and stained with 1% toluidine blue. Subsequently, the area of interest was trimmed, and ultrathin sections (0.06 µm) were obtained with a diamond knife. These sections were then stained with lead citrate and examined under a JEM1010 Jeol electron microscope equipped with a 4 Kx4K TemCam-F416 Digital camera. All the images were obtained at ×15,000 magnification. In order to analyze the afferent synapses of the hippocampal granule neurons, 30 images per animal containing the molecular layer (ML) were obtained. With respect to the ultrastructural organization of the efferent synapses of these neurons, at least 30 images per animal containing the *stratum lucidum* and the inner boundary of the *stratum pyramidale* of the CA3 region were obtained.

The density of synapses (number of synapses per µm^2^) and the area of the postsynaptic density (PSD) were measured in each sub-region separately. In addition, the density of presynaptic vesicles in the presynaptic terminal (number of vesicles per µm^2^) and the number of vesicles fused with the active zone were measured. Given that some presynaptic terminals were not fully included in the image due to the high complexity and size of these structures, a circular region of interest was used to count the number of vesicles and to calculate their density. The different parameters were measured manually in the images using ImageJ software. The density of synapses was calculated by dividing the total number of synapses in each image by the known area of the image. 100–150 synapses per experimental group and region were analyzed.

### Volume estimation of the dentate gyrus

To measure the DG volume, we used a semi-automatic Cavalieri system (ImageJ v.1.47, NIH, USA, http://rsbweb.nih.gov/ij/) in a series of 50-µm sections stained with DAPI, as previously described.

### Cell counts

The total number of mature granule neurons was calculated under a LSM710 Zeiss confocal microscope (×63 oil immersion objective) using the physical dissector method adapted for confocal microscopy^23^. Briefly, five stacks of images per animal were used to determine the density of mature granule neurons in a reference structure of known volume (30 (*X*) × 30 (*Y*) × 10 (*Z*) μm). The average density of these cells was then multiplied by the total volume of the DG in order to calculate the total number of mature granule neurons per animal^38^.

The total number of DCX^+^ neuroblasts was calculated using the physical dissector method adapted for confocal microscopy (Zeiss LSM710), as previously described^38^. Six stacks of images randomly selected from one series of slices were examined per animal.

The total number of IdU^+^ and fractin^+^ cells was counted under an optical fluorescence microscope (Zeiss Axioskop2) using the optical-dissector method, as previously described^39^.

In order to study the maturation of newborn neurons, the percentage of 8-week-old RFP^+^ newborn neurons that expressed NeuN was analyzed in ×60 magnification (2× zoom) confocal images. A minimum of 100 cells per experimental condition were examined.

### Colocalization between Cy5 signal and MAP-2

Colocalization between Cy5 and MAP-2 was measured in the DG of stereotaxically injected mice. For this purpose, 16-bit confocal images of the DG (×60 oil objective, 2× zoom) were obtained under a Zeiss LSM710 confocal microscope. Five images per animal were acquired. Images were analyzed by means of the JaCoP colocalization plugin for ImageJ^40^. Briefly, images were subjected to an invariant threshold. The plugin analyzed the colocalization area above the threshold between the Cy5^+^ or MAP-2^+^, and Mander´s coefficients were calculated and shown in the graphs.

### Morphometric analysis of newborn granule neurons

For immunohistochemical analysis, series of 50-µm brain sections were made up randomly of one section from every ninth. Three series of sections from each animal were used for the immunohistochemical detection of RFP. Fifty randomly selected neurons from each experimental condition were reconstructed under a LSM710 Zeiss confocal microscope (×25 oil immersion objective). Confocal stacks of images were obtained (*Z*-axis interval: 2 µm), and Z-projections were analyzed in order to determine total dendritic length and degree of dendritic arbor branching (Sholl´s analysis). All cells were traced using *NeuronJ* plugin for ImageJ software. Sholl's analysis was performed using the plugin *ShollAnalysis* for ImageJ. This analysis consists of placing a central point on the cell soma and tracing concentric circles (separated by a distance interval of 10 µm). The number of times that the dendritic tree intersected each circle is shown in the graphs (number of crossings). The percentage of cells aberrantly showing several primary apical dendrites was calculated as previously described^23^. In addition, in order to measure the migration of retrovirus-labeled newborn granule neurons, the distance between the hilar border of the subgranular zone and the center of the cell nucleus of each RFP^+^ cell was measured as previously described^41^. One hundred cells per genotype were analyzed.

### Morphometric analysis of the dendritic spines of newborn granule neurons

Dendritic spines were analyzed in 8-week-old retrovirus-labeled newborn neurons. Confocal stacks of images were obtained in a LSM710 Zeiss confocal microscope (×63 oil immersion objective; *XY* dimensions: 67.4 μm; *Z*-axis interval: 0.2 µm). The dendritic length of each segment was measured on Z-projections, and the number of dendritic spines was counted using the *NeuronStudio* software (CNIC, Mount Sinai School of Medicine, 2007–2009)^42^. Prior to spine analysis, images were deconvoluted using the Huygens Professional software (Scientific Volume Imaging). A minimum of 100 dendrites per experimental group were examined. Dendritic fragments were automatically constructed using *NeuronStudio* software, and then individual seed points were rectified manually to more accurately trace the dendrite. Thereafter, the dendritic spines were detected by the software and assigned to one of the following three categories: stubby, thin, and mushroom, as previously described^3,41^. Each spine was checked manually in order to assure accurate classification. The total density of spines (number of spines/µm), the number and percentage of each type of spine, the spine head diameter, and the approximate spine length (Max-DTS) were calculated for each type of spine, as previously described^41^.

### Area of mossy fiber terminals of newborn granule neuronsThe abbreviation MFT has been deleted from the heading and introduced in the text, please confirm.OK

The area of individual mossy fiber terminals (MFTs) was measured in the CA3 region of RFP-injected animals. A minimum of 20 stacks of images per experimental condition were obtained in a LSM710 Zeiss confocal microscope (×63 oil immersion objective; *XY* dimensions: 100 µm; Z-interval: 0.5 µm). Stacks were randomly obtained from the sections comprising the series. Z-projections were obtained, and the area of each MFT was measured manually using ImageJ software, as previously described^3,43^. A minimum of 100 MFTs per experimental condition were measured.

### Statistical analysis

Statistical analysis was performed using the SPSS 23 software (SPSS, 1989; Apache Software Foundation, Chicago, IL, USA). The Kolmogorov–Smirnov test was used to test the normality of the sample distribution. Data were analyzed by a Student’s *t*-test in the case of normal sample distribution, or by a nonparametric test (Mann–Whitney *U* test) in those cases in which normality could not be assumed. Graphs represent mean values ± SEM. The analysis of the percentage of the types of spine was accomplished by a chi-squared (*χ* ^2^) test.

## Results

### The presence of Tau-Cy5 does not cause significant alterations in gross anatomy of the DG

A schematic representative diagram of the experimental design is shown in Fig. 1a. Briefly, animals were subjected to their respective stereotaxic surgeries and 8 weeks later sacrificed. Figure 1b shows representative tile-scan images of the whole hippocampal region of animals injected with either PBS-Cy5 or Tau-Cy5. Tau-Cy5-injected animals showed an intense Cy5 signal at this time point, whereas this signal was mostly absent in mice injected with PBS-Cy5. In order to confirm the soluble nature of Tau, an analytic ultracentrifugation assay was performed. This analysis showed that injected Tau was composed mostly of monomers (54%) and dimers (31.4%) (Supplementary Figure S1). The presence of intracellular soluble Tau in granule neurons was studied by measuring the colocalization between Cy5 signal and MAP-2 staining in the DG. As shown in Supplementary Figure S2A,B, an increased signal of Cy5 colocalizing with MAP-2 staining was observed in Tau-Cy5-injected animals in comparison to PBS-Cy5-injected ones (*U* = 1000; *p* = 0.009). These observations thus reveal the presence of intracellular Tau in the somatodendritic compartment of these cells. Mander´s coefficients are shown in Supplementary Figure S2B. To rule out the possibility that the stereotaxic injection of soluble Tau triggers massive neurodegeneration of the region, we measured the volume of the DG in both animal groups. No changes in this parameter were observed (*t* = 0.332; *p* = 0.745) (Fig. 1c). In order to rule out the possibility that soluble Tau injections trigger the generalized apoptosis of granule neurons, the number of apoptotic fractin^+^ cells was calculated in Tau-Cy5- and PBS-Cy5-injected animals (*U* = 5.000; *p* = 0.486) (Fig. 1d). In addition, the total number of mature granule neurons remained unaltered after Tau-Cy5 injection (*t* = 0.702; *p* = 0.493) (Figs. 1e, f).

To further characterize the effects of soluble Tau injection on the general rate of AHN, we counted the number of DCX^+^ neuroblasts, finding no significant changes in this parameter (*t* = 0.524; *p* = 0.609) (Supplementary Figure 3A,B). Moreover, we studied the long-term survival of newborn granule neurons by quantifying the number of 12-week-old IdU^+^ cells in the DG (Supplementary Figure 3C). No changes in this parameter were found (*U* = 4.000; *p* = 0.629). Given the absence of changes in the aforementioned parameters, we conclude that the presence of soluble Tau-Cy5 does not cause dramatic alterations in the gross anatomy of the DG or in the general rate of AHN.

### Tau-Cy5 impairs the connectivity of granule neurons at the ultrastructural level

**Fig. 2:**
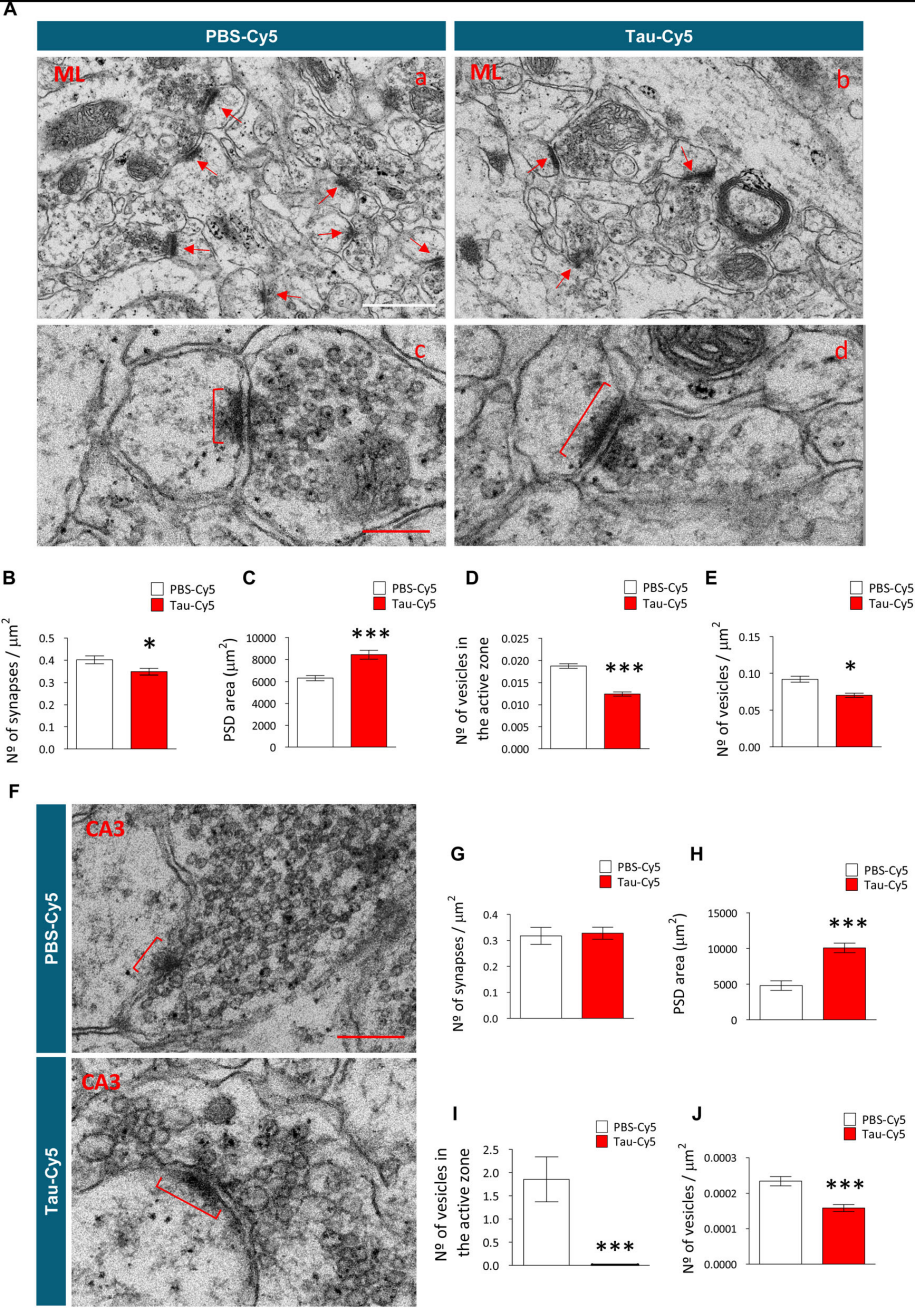
Tau-Cy5 impairs the connectivity of granule neurons at the ultrastructural level (**a**) Representative electron microscopy images of the afferent synapses of granule neurons in the molecular layer (ML) of PBS-Cy5- and Tau-Cy5-injected animals, showing their density (a, b) and their morphometric features (c, d). (**b–e**) Quantification of the density of synapses (**b**), postsynaptic density (PSD) area (**c**), number of synaptic vesicles fused to the active zone (**d**), and density of vesicles in the presynaptic terminal (**e**) in the ML of PBS-Cy5- and Tau-Cy5-injected animals. (**f–j**) Representative electron microscopy images (**f**) and quantification of the density (**g**) and morphometric features (**h–j**) of the efferent synapses of granule neurons located in the hippocampal CA3 region of PBS-Cy5- and Tau-Cy5-injected animals. Tau-Cy5 injection led to alterations in the density and ultrastructure of both afferent and efferent synapses of granule neurons. In (**b–e**) and in (**g–j**) graphs represent mean ± SEM; *n* = 3 mice per experimental condition; *0.05 > *p* ≥ 0.01, ***p < 0.001 (Student´s *t*-test or Mann–Whitney *U* test). Arrows indicate synapses. Brackets indicate PSDs. White scale bar: 600 nm. Red scale bar: 200 nm

To study whether the stereotaxic injection of soluble Tau in the DG causes long-term alterations in the synapses of granule neurons, we analyzed the afferent (Figs. 2a–e) and efferent (Figs. 2f–j) synapses of these cells at the ultrastructural level in the ML of the DG and in the CA3 hippocampal regions, respectively. Figure 2a shows representative electron microscopy images of the ML of animals injected with either PBS-Cy5 or Tau-Cy5. In this region, stereotaxic injection of soluble Tau caused a reduction in the density of synapses (Fig. 2b) (*U* = 3998.5; *p* = 0.044). Moreover, the area of the PSDs of the remaining synapses increased (Fig. 2c) (*U* = 2399; *p* < 0.001), whereas both the number of synaptic vesicles fused to the active zone (Fig. 2d) (*t* = 6.282; *p* < 0.001) and the density of vesicles in the presynaptic terminal (Fig. 2e) (*U* = 170; *p* = 0.028) decreased in animals injected with Tau-Cy5. Figure 2f shows representative images of the CA3 region of animals injected with either PBS-Cy5 or Tau-Cy5. Stereotaxic injection of soluble Tau did not alter the density of synapses (Fig. 2g) (*U* = 2285.5; *p* = 0.419) but did lead to an increase in the area of the PSDs (Fig. 2h) (*U *= 1230; *p* < 0.001) and to a marked reduction in both the number of synaptic vesicles fused to the presynaptic active zone (Fig. 2i) (*U* = 337; *p* = *p* < 0.001) and the density of synaptic vesicles in the presynaptic terminal (Fig. 2j) (*t* = 4.553; *p* < 0.001). In summary, stereotaxic injection of soluble Tau triggered alterations in the number and ultrastructure of synapses of the general population of granule neurons.

### Tau-Cy5 alters the morphology of newborn granule neurons

**Fig. 3:**
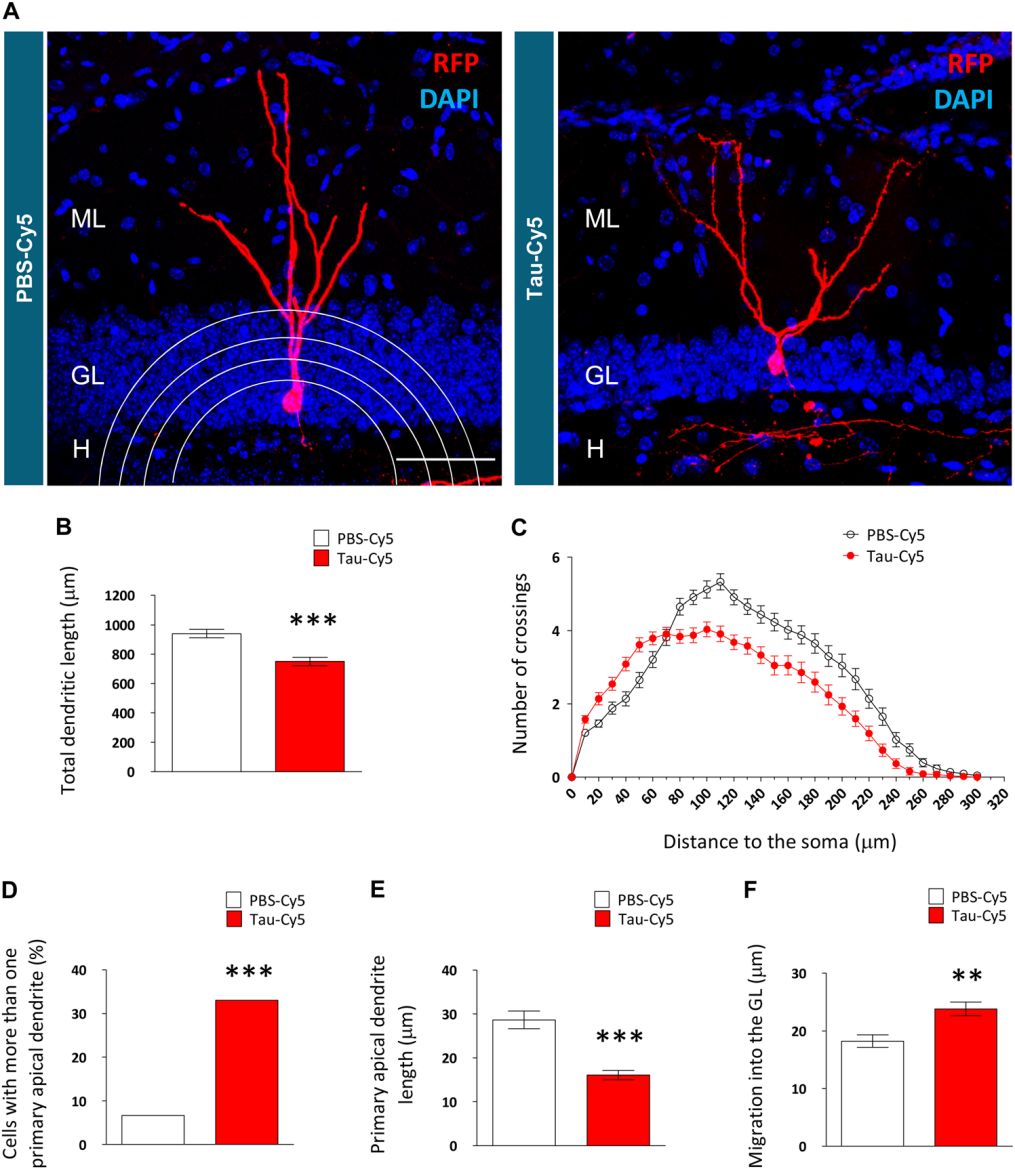
The presence of Tau-Cy5 alters the morphology of newborn granule neurons (**a**) Representative images of RFP-labeled 8-week-old newborn granule neurons in PBS-Cy5- and Tau-Cy5-injected mice. A schematic representation of Sholl's analysis is shown in the image corresponding to a PBS-Cy5-injected mouse. (**b–f**) Quantification of total dendritic length (**b**), Sholl's analysis (**c**), percentage of neurons with several primary apical dendrites (**d**), length of the apical dendrite (**e**), and migration into the GL (**f**) of newborn granule neurons in PBS-Cy5- and Tau-Cy5-injected mice. Tau-Cy5 injection altered the morphology and migration of newborn granule neurons in the DG. ML, molecular; GL, granular layer; H, hilus. In (**b**, **c**, **e** and **f**) graphs represent mean ± SEM; **0.01 > *p* ≥ 0.001, ***p < 0.001 (Student´s *t*-test or Mann–Whitney* U* test). Graph in (**d**) represent percentage; ***p < 0.001 (*χ* ^2^ test). *N* = 4 mice per experimental condition. Scale bar: 50 µm

To study whether soluble Tau has an additional impact on the morphology of newly generated granule neurons, we injected an RFP-expressing retrovirus into the DG of animals injected with PBS-Cy5 or Tau-Cy5 (Fig. 1a). After the complete maturation of these cells (8 weeks post-injection), the animals were sacrificed. To ensure that the cells analyzed were at the same developmental stage in PBS-Cy5- and Tau-Cy5-injected animals, the percentage of RFP^+^ neurons that expressed the mature neuron marker NeuN was calculated. The vast majority of cells were NeuN^+^ in both experimental groups (this percentage was 94% in the case of PBS-Cy5-injected animals and 97% in the case of Tau-Cy5-injected mice) (Supplementary Figure S3C **)**. These observations thus indicate that the cells analyzed were at the same developmental stage. Morphometric determinations were then performed in RFP-labeled newborn granule neurons. Figure 3a shows representative images of the morphology of newborn granule neurons of the two groups of animals. Stereotaxic injection of soluble Tau caused a reduction in the total dendritic length of newborn granule neurons (Fig. 3b) (*t* = 4532; *p* ≤ 0.001) and altered the morphology of the whole dendritic tree, as revealed by Sholl´s analysis (Fig. 3c). Statistical comparisons of Sholl´s analysis corresponding to each 10-μm length dendritic segment are shown in Supplementary Table 1. According to these morphological alterations, the percentage of newborn granule neurons showing more than one primary apical dendrite increased (Fig. 3d) (*χ* ^2^ = 17.828; *p* ≤ 0.001) and the length of the primary apical dendrite decreased (Fig. 3e) (*U* = 1763; *p* ≤ 0.001) in Tau-Cy5-injected animals. In addition, the migration of these neurons into the GL increased (Fig. 3f) (*U* = 2522; *p* = 0.006).

### Tau-Cy5 impairs the connectivity of newborn granule neurons

**Fig. 4:**
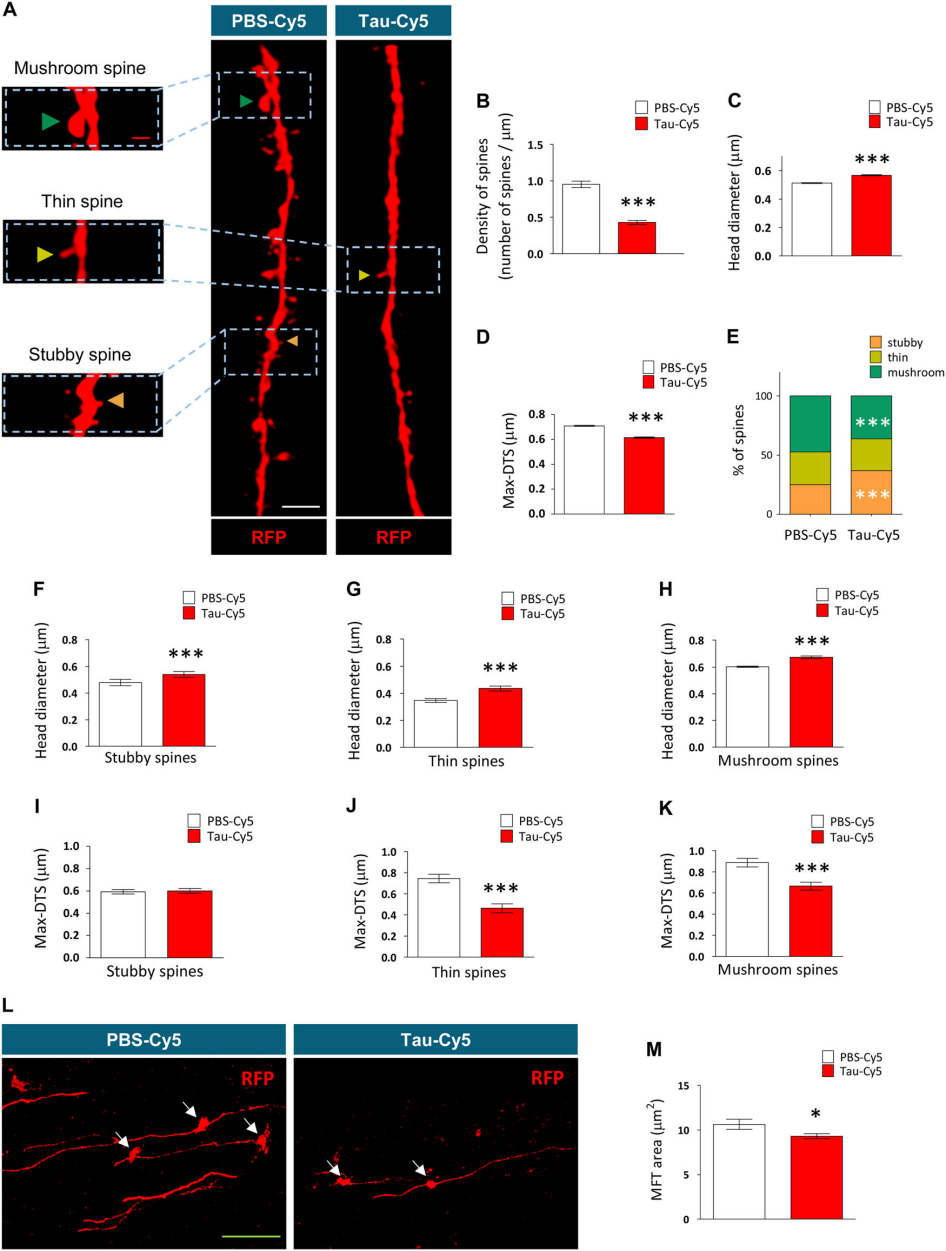
Tau-Cy5 impairs the connectivity of newborn granule neurons (**a**) Representative images of 8-week-old RFP-labeled newborn granule neuron dendrites of PBS-Cy5- and Tau-Cy5-injected mice showing their dendritic spines. The distinct spine morphologies—stubby (orange triangles), thin (yellow triangles), and mushroom (green triangles)—are shown in high-power magnifications. (**b–d**) Quantification of the density of dendritic spines (**b**), spine head volume (**c**) and spine Max-DTS (**d**) of newborn granule neurons of PBS-Cy5- and Tau-Cy5-injected mice. Tau-Cy5 injection decreases the density of dendritic spines and alters their morphology. (**e**) Quantification of the percentages of stubby, thin and mushroom spines in newborn granule neurons of PBS-Cy5- and Tau-Cy5-injected mice. Tau-Cy5 injection altered the proportion of the different types of spines, decreasing that of mushroom spines and increasing that of stubby ones. (**f**–**h**) Quantification of the head diameter of stubby (**f**), thin (**g**) and mushroom (**h**) dendritic spines of newborn granule neurons of PBS-Cy5- and Tau-Cy5-injected mice. (**i–k**) Quantification of the Max-DTS of stubby (**i**), thin (**j**) and mushroom (**k**) dendritic spines of newborn granule neurons of PBS-Cy5- and Tau-Cy5-injected mice. Tau-Cy5 injection increased the head diameter of every type of spine and decreased the Max-DTS of thin and mushroom ones. (**l**,**m**) Representative images (**l**) and quantification (**m**) of the newborn neuron MFT area in the CA3 region of PBS-Cy5- and Tau-Cy5-injected mice. Tau-Cy5 injection reduced the area of newborn neuron MFTs. Graphs in (**b–d**, **f–k**,** m**) represent mean ± SEM; *0.05 > *p* ≥ 0.01, ***p < 0.001 (Mann–Whitney U test). Graph in (**e**) represent percentages. ***p < 0.001 (χ2 test). *n* = 4 mice per experimental condition. White scale bar: 3 µm. Red scale bar: 1 µm. Green scale bar: 20 µm

To address whether the stereotaxic injection of soluble Tau causes long-term effects on the connectivity of adult-born granule neurons, we studied the number, size and subtypes of dendritic spines of RFP-labeled newborn granule neurons of animals injected with PBS-Cy5 or Tau-Cy5 (Fig. 4). Figure 4a shows representative images of dendritic fragments of newborn granule neurons of animals injected with PBS-Cy5 or Tau-Cy5. Stereotaxic injection of soluble Tau reduced the density of dendritic spines (Fig. 4b **)** (*U* = 8153; *p* ≤ 0.001). In addition, the remaining spines showed an increase in head volume (Fig. 4c) (*U* = 10472778; *p* ≤ 0.001), whereas the approximate measurement of spine length (Max-DTS) decreased (Fig. 4d) (*U* = 10619616.5; *p* ≤ 0.001). According to morphological criteria, dendritic spines are classified into stubby, thin and mushroom subtypes^41^. Figure 4e shows that the stereotaxic injection of soluble Tau caused a marked increase in the percentage of stubby spines (*χ* ^2^ = 73.036; *p* ≤ 0.001), whereas the percentage of mushroom spines was drastically reduced (*χ* ^2^ = 52.761; *p* ≤ 0.001). Next, we studied various morphometric parameters of each spine type separately (Figs. 4f–k). Tau-Cy5 increased the head volume of each spine type (stubby **(**Fig. 4f) (*U* = 204,728; *p* ≤ 0.001), thin (Fig. 4g) (*U* = 139,626; *p* ≤ 0.001) and mushroom (Fig. 4h **)** (*U* = 376,153; *p* ≤ 0.001)) and reduced the Max-DTS of thin (Fig. 4j) (*U* = 75,118; *p* ≤ 0.001) and mushroom (Fig. 4k) (*U* = 256,459; *p* ≤ 0.001) spines. Finally, the area of the axonal terminals (mossy fiber terminals, MFTs) of newborn granule neurons was measured in the CA3 hippocampal subfield of PBS-Cy5- and Tau-Cy5-injected animals. Tau-Cy5 injection reduced the area of MFTs (*U* = 7700.5; *p* = 0.03) (Figs. 4l, m **)**.

### Tau-Cy5 impairs behavioral pattern separation

**Fig. 5:**
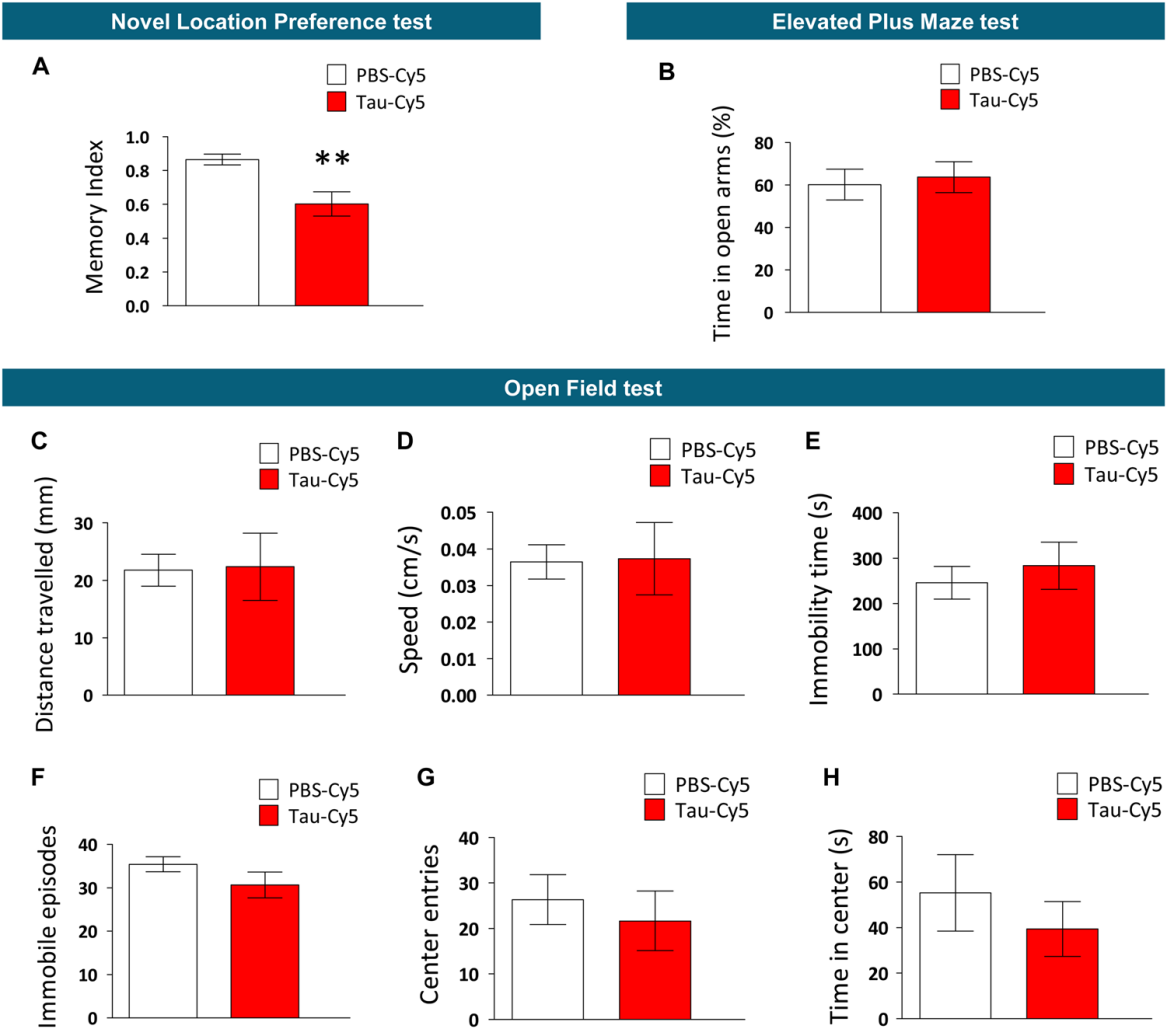
Tau-Cy5 selectively alters behavioral pattern separation ability but does not cause alterations in the general behavior of the animals PBS-Cy5- and Tau-Cy5-injected mice were subjected to pattern separation, motor skills and anxiety-like-related behavioral tests. (**a**) Memory index (time spent exploring the object placed in the new location/total time of exploration) in the Novel Location Preference test. Tau-Cy5 injection impaired pattern separation skills of the mice. (**b**) Percentage of time spent in the open arms of the Elevated Plus Maze test. Tau-Cy5 injection did not affect anxiety-like behavior. (**c**–**h**) Total distance traveled (**c**), speed (**d**), immobility time (**e**), number of immobile episodes (**f**), and number of entries (**g**) and time (**h**) in the center of the arena in the Open Field test. Tau-Cy5 injection did not alter the anxiety-like and motor behavior of the mice. Graphs represent mean ± SEM; *n* = 9 mice per experimental condition; **0.01 > *p* ≥ 0.001 (Student's *t*-test)

Newborn granule neurons play a relevant role in hippocampal-dependent memory^18,44,45^, and they are particularly important for behavioral pattern separation skills^24^. Given the alterations observed in the connectivity and the morphology of these cells, we questioned whether behavioral pattern separation capacity was also impaired in the mice. The novel location preference (NLP) test is considered a reliable model of pattern separation in rodents^37,46,47^. The stereotaxic injection of Tau-Cy5 reduced the percentage of time spent exploring the object placed in a new location (Memory index, *t* = 3.364; *p* = 0.007), thus indicating impaired behavioral pattern separation ability in these mice (Fig. 5a). However, we questioned whether these alterations could be due to either anxiety-like behavior or motor impairment of these animals. To explore these possibilities, we performed Elevated Plus Maze (EPM) and Open Field (OF) tests respectively. Soluble Tau-Cy5 did not alter anxiety-like behavior in the EPM test, as Tau-Cy5-injected animals displayed a percentage of time in open arms comparable to that of their PBS-Cy5-injected counterparts (*t* = −0.339; *p* = 0.739) (Fig. 5b). In addition, the behavior of the mice in the OF test remained unaltered after the stereotaxic injection of Tau-Cy5. Soluble Tau did not change parameters related to motor behavior, such as the total distance traveled (Fig. 5c) (*t *= −0.091; *p* = 0.929), mean speed (Fig. 5d) (*t* = −0.076; *p* = 0.940), time immobile (Fig. 5e) (*t* = −0.595; *p* = 0.561) and number of immobile episodes (Fig. 5f) (*t* = 1.370; *p* = 0.195). In addition, it did not alter those related to anxiety-like behavior, such as the number of entries to the center (Fig. 5g) (*t* = 0.545; *p* = 0.593) or the time spent in the center of the arena (Fig. 5h) (*t* = 0.765; *p* = 0.458). On the basis of these observations, we can conclude that the impairment in the behavioral pattern separation skills of the mice injected with Tau-Cy5 was specific and not due to a non-selective alteration in their general behavior.

## Discussion

Tau is a neuronal MAP that plays numerous physiological roles^48^. In recent decades Tau functions were believed to be limited to the axonal compartment^49–54^. However, growing evidence supports the relevance of Tau also in the neuronal somatodendritic compartment^3,5,55^. In this regard, we have recently shown that Tau is required for *in vivo* afferent synapse formation in newborn granule neurons^3^. Although the absence of Tau has been reported to confer neuroprotection, we have also shown that this MAP is required for the stimulatory effects of certain external stimuli on AHN^3^. Thus, the current belief in the field is that a tight regulation of Tau metabolism is required for each neuron to achieve the appropriate balance between stability and plasticity needed at any given time. In line with the aforementioned hypothesis, post-transcriptional changes occurring in Tau protein have drastic consequences on its physiological functions. For instance, Tau phosphorylation reduces its affinity to bind microtubules, which leads to microtubule destabilization and cytoskeleton instability^56–61^. Although this instability may occur in neurons during discrete periods of time in which a higher degree of plasticity is required, chronic dysregulation of Tau phosphorylation has severe effects on neuronal function^57,62,63^. An important point to bear in mind is the link between Tau phosphorylation and the capacity of this protein to form insoluble aggregates^10,64^. On the one hand, phosphorylation can induce Tau aggregation *in vitro*, probably due to the addition of negative charges^65,66^. Moreover, it is also believed that Tau detachment from microtubules can also trigger the aggregation of this protein by allowing greater interaction between phosphorylated and non-phosphorylated Tau monomers^65,66^. Importantly, growing evidence suggests that Tau oligomers are also highly toxic for neurons, and a prion-like mechanism has been proposed to explain the spread of Tau from neuron to neuron during neurodegenerative processes^11^. In the present work, we aimed to address the effects of non-aggregated, soluble Tau on the structural plasticity of the granule neurons of the DG. Despite the soluble nature of the Tau-Cy5 used in this study^67^, we have demonstrated a substantial *in vivo* detrimental effect caused by the exposure of granule neurons to this soluble protein. These neurons showed markedly decreased connectivity (reduced number of synapses) in the ML. It should be noted that the synaptic contacts with the perforant pathway occurring in the ML are the main afferent connections received by granule neurons from the Entorhinal cortex. Hence, these contacts are critical for the synaptic integration of granule neurons in the trisynaptic circuit^68–72^. Our data show that the phenomenon of reduced afferent connectivity also occurs in newborn granule neurons, which exhibited a reduced number of dendritic spines. Interestingly, this reduction is attributable mainly to the specific reduction of mushroom spines, as the number of thin spines remained unchanged while stubby spines increased in number. Of note, mushroom spines are considered to harbor the strongest synapses in the central nervous system, whereas thin and stubby spines are thought to be more plastic and to harbor weaker or more immature synapses^3,73^. Hence, the marked decrease in the number of mushroom spines, together with the increase in the proportion of stubby spines, may lead to decreased synaptic efficiency and instability of the global network at this level. Interestingly, at the ultrastructural level, synapses in the ML displayed a higher PSD area, which is in agreement with the increase observed in the head volume of dendritic spines of newborn granule neurons. However, given the marked depletion of synaptic vesicles at the presynaptic level and the reduced number of synapses, uncoupling between the presynaptic and the postsynaptic elements may occur in the ML of Tau-Cy5-injected animals. Alternatively, the increased PSD area and spine head volume could be interpreted as a compensatory mechanism through which to counteract the reduced synaptic transmission caused by soluble Tau. However, despite this putative attempt to compensate for reduced synaptic efficiency, the final outcome of the scenario is a clear impairment in behavioral pattern separation ability. In agreement with the proposed decrease in synaptic transmission, it should be mentioned here that the area of the MFTs was reduced by soluble Tau, thus supporting the notion that newborn granule neurons provide an insufficient contribution to the hippocampal trisynaptic circuit.

In line with this, it has been proposed that newly born neurons contribute to the creation of memory representations in the hippocampal network by enhancing pattern separation^24,26^. Although the specific contribution of both developmentally generated and newly born granule neurons to the behavioral impairment cannot be evaluated separately in the present paradigm, it can be hypothesized that the alterations in structural plasticity in Tau-Cy5-injected animals are, to some extent, responsible for the impaired capacity of these animals to perform behavioral pattern separation.

In addition, another relevant aspect believed to be critical for newborn granule neuron functionality is their morphology^74^. In this regard, certain pathological conditions trigger the appearance of a particular morphological phenotype in newborn granule neurons (namely, the “V-shape” phenotype)^23,74^. Whereas the normal phenotype of these neurons (the “Y-shape”) is characterized by the presence of a single primary apical dendrite extensively branched in the ML, “V-shape” newborn granule neurons display several primary apical dendrites emerging from the soma and a markedly reduced distal branching of the dendritic tree^23^. Interestingly, although the causal relationship of these morphological alterations and behavioral impairments has not been established to date, they occur in parallel in diverse animal models of various pathologies^74^. Importantly, the aforementioned “V-shape” phenotype of granule neurons has been observed in the brains of AD patients^23^. Here we show that these morphological features appear in newborn granule neurons exposed to soluble Tau. Whether or not these morphological alterations also contribute to the impairment of behavioral pattern separation skills is an open question that requires further attention.

Noteworthy, increased levels of intracellular Tau are observed in the brains of AD patients^75^. This increase in intracellular Tau is toxic for neurons while a reduction is neuroprotective in animal models of neurodegenerative disorders^76^. Various mechanisms triggered by soluble Tau could be responsible for the alterations in the plasticity of granule neurons observed in the present study^77,78^. On the one hand, microtubule destabilization may have a direct impact on granule neuron morphology^3,79^, but it may also contribute to the impairment of axonal and dendritic transport of mitochondria and other cargoes to synaptic sites^4,77,78^. In this regard, we have previously demonstrated that Tau plays a crucial role in regulating mitochondrial axonal transport in hippocampal neurons^4^. This deficient delivery of mitochondria may have important consequences for granule neuron connectivity^80^. In addition, despite the soluble nature of the extracellular Tau injected, a remote possibility of intracellular aggregation cannot be completely ruled out. In this regard, it is also known that excess intracellular Tau activates the unfolded protein response by impairing endoplasmic-reticulum-associated degradation of several proteins, including Tau itself^81^. Although Tau has been described to enter nerve cells via several mechanisms^67,82,83^, our group has shown that a principal mechanism of Tau entry into neurons includes the stimulation of type M1 and M3 muscarinic acetylcholine receptors^84–87^. Noteworthy, these are the most abundant muscarinic cholinergic receptors expressed in the hippocampus. Tau interaction with these receptors (which are intracellularly coupled to G-proteins belonging to the G_q_/G_11_ family) triggers calcium entry into the neuron, and, consequently, neuron depolarization^88^. Excessive depolarization is toxic for neurons and may contribute to the additional release of Tau to the extracellular space—a mechanism that has been proposed to exacerbate damage through neuron-to-neuron spread throughout the brain.

Nevertheless, here we demonstrate, for the first time, that stereotaxic injection of soluble Tau has detrimental long-term effects on the structural plasticity and connectivity of hippocampal granule neurons. Furthermore, we show that these alterations occur in parallel to a considerable impairment of behavioral pattern separation ability.

## Electronic supplementary material

Legends to Supplementary Figures

Supplementary Table 1

Supplementary Figure S1

Supplementary Figure S2

Supplementary Figure S3
