# Supplementary material for: Soluble Tau has devastating effects on the structural plasticity of hippocampal granule neurons
Source: Transl Psychiatry. 2017 Dec 8;7:1267. doi: 10.1038/s41398-017-0013-6 (PMC5802513; doi:10.1038/s41398-017-0013-6)
Supplement: Supplementary file 1 — Legends to Supplementary Figures [file 41398_2017_13_MOESM1_ESM.docx]

**Supplementary Figure S1. Analysis of Tau by ultracentrifugation. (A)** Graph shows the peak profile obtained by Tau sedimentation analysis. The Continuous distribution c (s) and sedimentation coefficient (S) of each peak are shown. The first peak (1.7S) is compatible with the theoretical mass of elongated Tau monomers, whereas the second peak (3.0S) is compatible with the theoretical mass of elongated Tau dimers. **(B)** Table shows the numeric values of the percentage (%) and S of each peak. Note that monomers are the most prevalent molecule in the Tau solution analyzed.

**Supplementary Figure S2. Colocalization between Cy5 signal and MAP-2 staining in the DG. (A)** Representative images and their high-power magnifications showing Cy5 (red) and MAP-2 (green) staining in the DG of PBS-Cy5- and Tau-Cy5-injected animals. **(B)** Quantification of the colocalization between Cy5 and MAP-2 staining in the DG. As shown, a marked colocalization can be observed in the case of Tau-Cy5-injected animals, in comparison to PBS-Cy5-injected ones. Mander´s coefficients are shown. GL, Granular layer; H, Hilus. Graphs represent mean ± SEM; **0.01 > p ≥ 0.001 (Mann-Whitney U test). n= 9 mice per experimental condition. Blue scale bar: 10 µm. Red scale bar: 50 µm.

**Supplementary Figure S3. Rate of adult hippocampal neurogenesis (AHN). (A)** Representative tilescan images of the DG showing DCX staining. (**B**) Quantification of the number of DCX^+^ neuroblasts in the DG. (**C**) Quantification of the number of 12-week-old IdU^+^ cells in the DG. (**D**) Percentage of 8-week-old RFP^+^ cells that expressed the mature neuron marker NeuN. As shown, no changes in these parameters occurred in response to soluble Tau injection. These data further support the notion that no massive neurodegeneration occurs as a result of soluble Tau injection in the DG. DG, Dentate gyrus; ML, Molecular layer; GL, Granular layer; H, Hilus. Graphs represent mean ± SEM. n= 100 cells per experimental condition. White scale bar: 100 µm. Yellow scale bar: 50 µm.
