# Supplementary material for: Soluble Tau has devastating effects on the structural plasticity of hippocampal granule neurons
Source: Transl Psychiatry. 2017 Dec 8;7:1267. doi: 10.1038/s41398-017-0013-6 (PMC5802513; doi:10.1038/s41398-017-0013-6)
Supplement: Supplementary file 2 — Supplementary Table 1 [file 41398_2017_13_MOESM2_ESM.docx]

| **Distance to the soma** | **Mann-Whitney U** | **p-value** |
| --- | --- | --- |
| **0-10 μm** | 1255.5 | 1 |
| **10-20 μm** | 866 | 0.003 |
| **20-30 μm** | 787 | 0.001 |
| **30-40 μm** | 800.5 | 0.002 |
| **40-50 μm** | 703.5 | ≤ 0.001 |
| **50-60 μm** | 775 | 0.001 |
| **60-70 μm** | 949 | 0.048 |
| **70-80 μm** | 1173 | 0.707 |
| **80-90 μm** | 843 | 0.006 |
| **90-100 μm** | 700.5 | ≤ 0.001 |
| **100-110 μm** | 731.5 | ≤ 0.001 |
| **110-120 μm** | 617 | ≤ 0.001 |
| **120-130 μm** | 631.5 | ≤ 0.001 |
| **130-140 μm** | 733.5 | ≤ 0.001 |
| **140-150 μm** | 797.5 | 0.002 |
| **150-160 μm** | 794.5 | 0.002 |
| **160-170 μm** | 883.5 | 0.016 |
| **170-180 μm** | 875 | 0.013 |
| **180-190 μm** | 871.5 | 0.012 |
| **190-200 μm** | 843 | 0.007 |
| **200-210 μm** | 844.5 | 0.007 |
| **210-220 μm** | 837 | 0.006 |
| **220-230 μm** | 864.5 | 0.005 |
| **230-240 μm** | 807 | 0.002 |
| **240-250 μm** | 845 | 0.002 |
| **250-260 μm** | 865 | ≤ 0.001 |
| **260-270 μm** | 958 | 0.001 |
| **270-280 μm** | 1079 | 0.021 |
| **280-290 μm** | 1106 | 0.044 |
| **290-300 μm** | 1161 | 0.186 |
| **300-310 μm** | 1168.5 | 0.102 |

**Supplementary Table 1.** Statistical comparisons of Sholl´s analysis corresponding to each 10-μm length dendritic segment of newborn granule neurons of animals injected with PBS-Cy5 or Tau-Cy5. Mann Whitney´s U test; n= 4 mice per experimental condition.
