# Supplementary figures and images for: Soluble Tau has devastating effects on the structural plasticity of hippocampal granule neurons
Source: Transl Psychiatry. 2017 Dec 8;7:1267. doi: 10.1038/s41398-017-0013-6 (PMC5802513; doi:10.1038/s41398-017-0013-6)

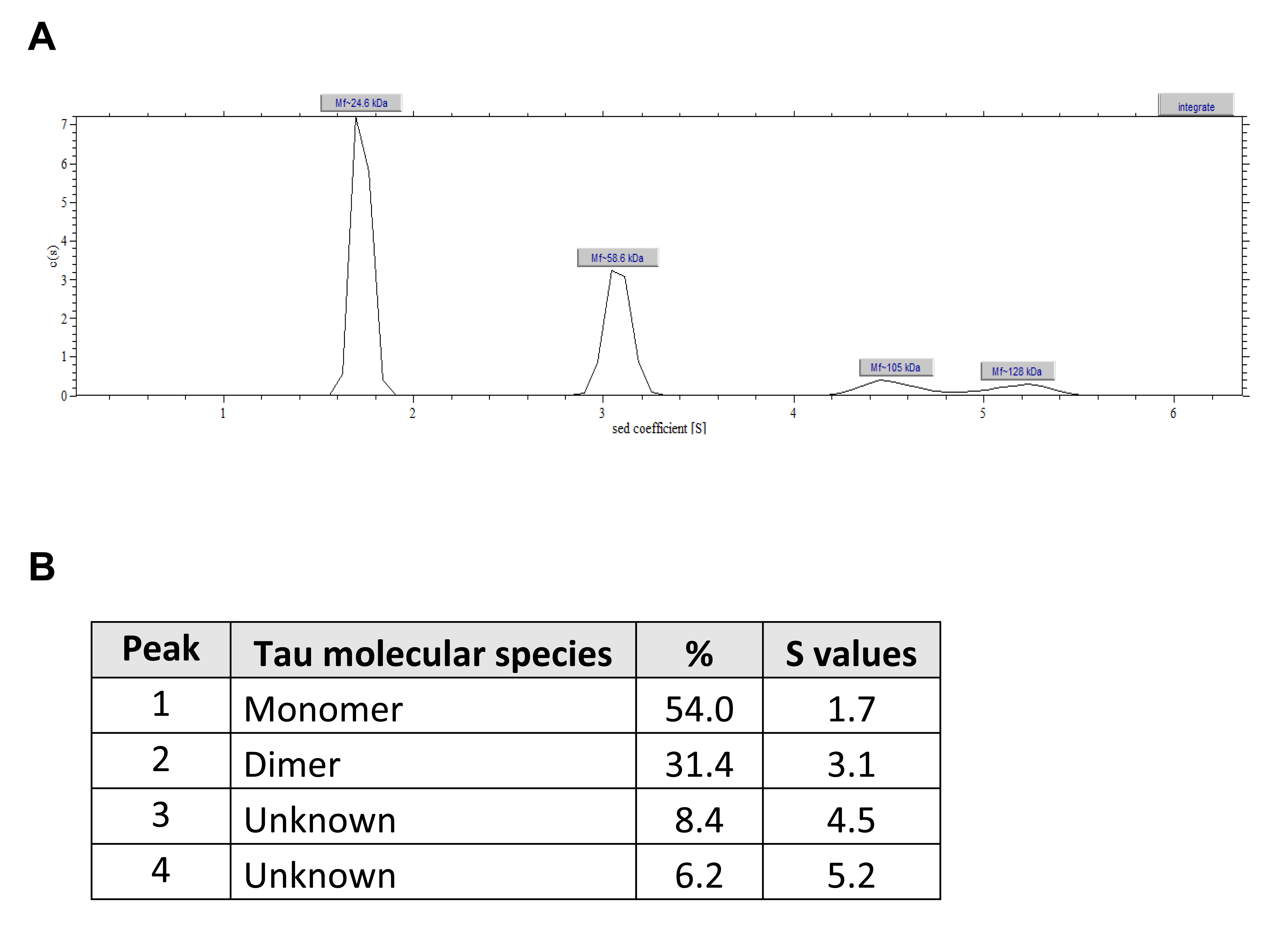

Supplement: Supplementary file 3 — Supplementary Figure S1 [file 41398_2017_13_MOESM3_ESM.tif]

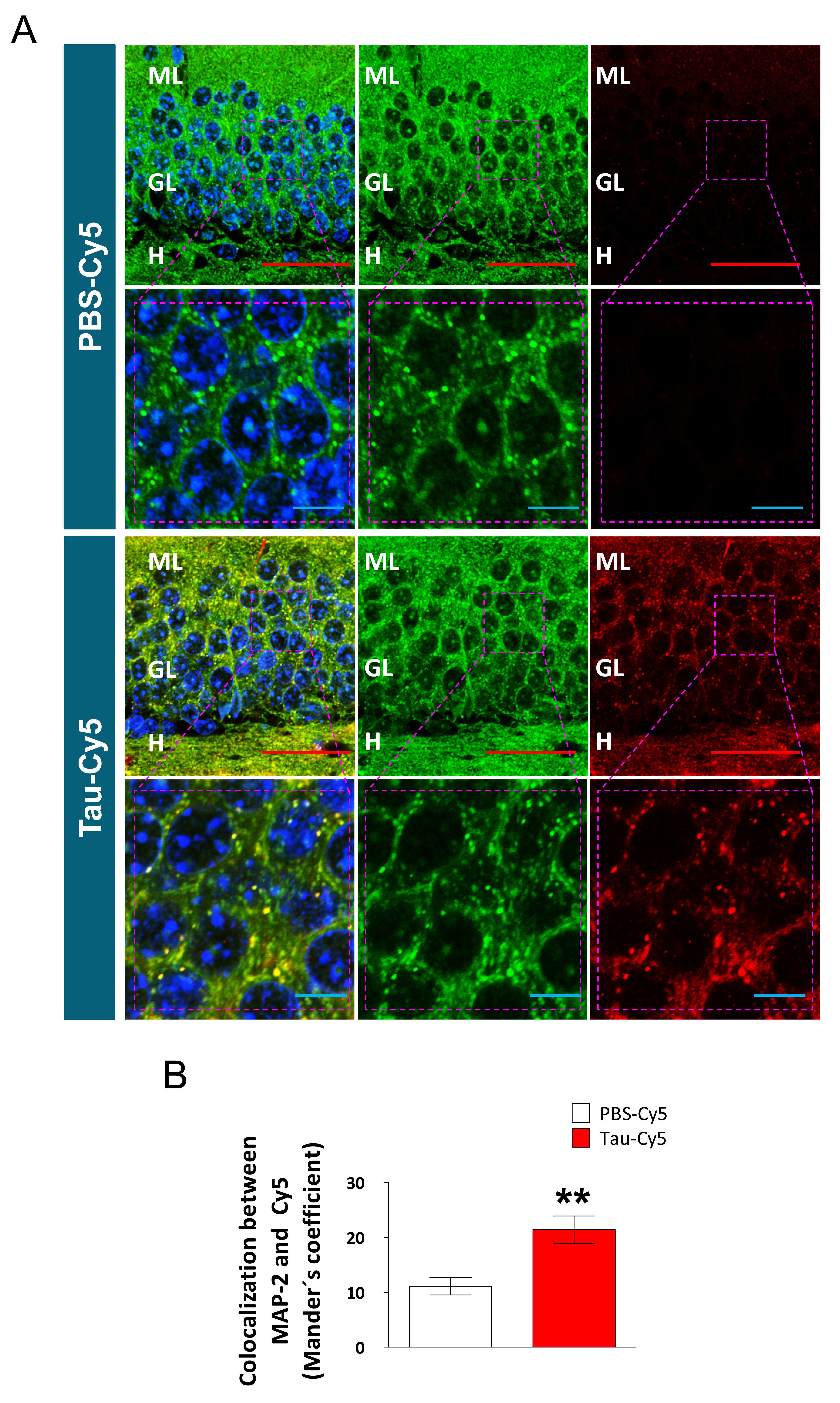

Supplement: Supplementary file 4 — Supplementary Figure S2 [file 41398_2017_13_MOESM4_ESM.tif]

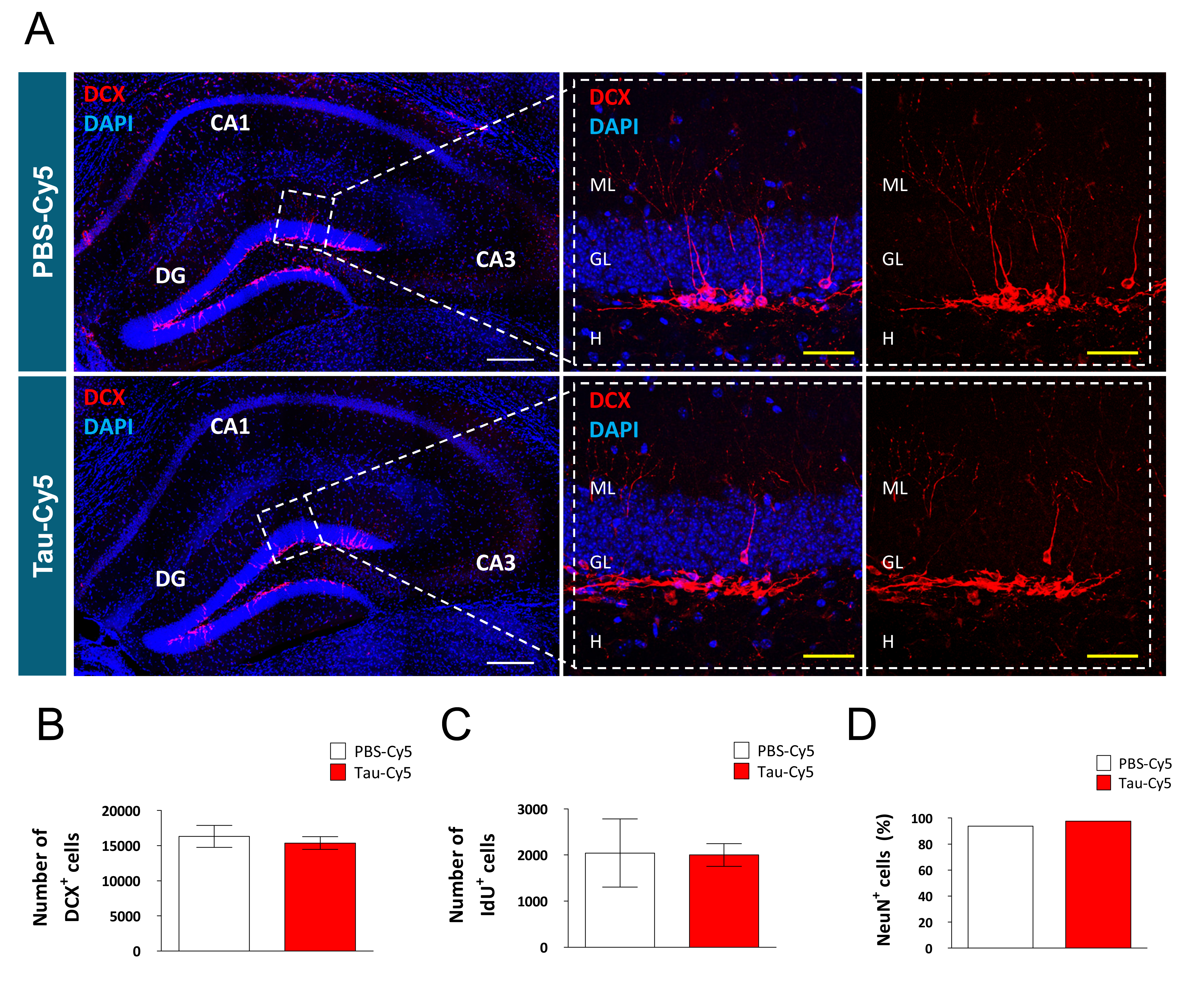

Supplement: Supplementary file 5 — Supplementary Figure S3 [file 41398_2017_13_MOESM5_ESM.tif]
